# Age and Sex Differences in the Prevalence of Specific Comorbidities among Patients with Pediatric Acute Lymphoblastic Leukemia and Lymphoblastic Lymphoma at Diagnosis

**DOI:** 10.1158/2767-9764.CRC-24-0517

**Published:** 2025-04-01

**Authors:** Xin Yang, Maua Mosha, Dave Bell, Jennifer Dean, Jennifer Mayer, Ernest K. Amankwah

**Affiliations:** 1 Clinical and Translational Science Institute of Southeastern Wisconsin, Medical College of Wisconsin, Milwaukee, Wisconsin.; 2 Institute for Clinical and Translational Research, Johns Hopkins All Children’s, St. Petersburg, Florida.; 3 Cancer and Blood Disorders Institute, Johns Hopkins All Children’s Hospital, St. Petersburg, Florida.; 4 Department of Oncology, Sidney Kimmel Comprehensive Cancer Center, Johns Hopkins University School of Medicine, Baltimore, Maryland.; 5 Department of Pediatrics, Johns Hopkins University School of Medicine, Baltimore, Maryland.

## Abstract

Limited knowledge exists on the prevalence of comorbidity among pediatric patients diagnosed with acute lymphoblastic leukemia (ALL) or lymphoblastic lymphoma (LL). To determine the prevalence of comorbidities present within 3 months before diagnosis among pediatric patients with ALL/LL and to examine if the prevalence varies by age, biological sex at birth, and race/ethnicity, we analyzed data of patients diagnosed with ALL/LL at ≤21 years of age from January 1, 2005, to June 30, 2020 (*n* = 5,455), using electronic health records data from the TriNetX Research Network database. Comorbidities examined included pulmonary, cardiac, cerebrovascular, vascular, developmental, immune, metabolic, infectious, genitourinary, digestive, muscle and connective tissue and central and peripheral nervous system conditions. Overall, the prevalence of comorbidity was 34.1% (*n* = 1,904), with significant differences observed based on sex and race/ethnicity. Females had a higher prevalence at 36.6% compared with males at 33.6% (*P* = 0.024). Similarly, non-Hispanic White patients had a higher prevalence of 37.5% compared with 33.1% in other racial/ethnic groups (*P* < 0.001). Analyses of specific comorbidities revealed notable differences in the prevalence of infectious diseases by biological sex at birth (female: 9.7%, male: 7.0%; *P* < 0.001) and digestive diseases by age at diagnosis (≤10 years: 13.8%, >10 years: 10.4%; *P* < 0.001). Although the overall prevalence of comorbidity at diagnosis showed minor differences across groups, disparities exist for specific comorbidities with females and younger patients having a higher prevalence of infectious diseases and digestive tract diseases, respectively. Future studies are needed to evaluate if these differences contribute to the disparities in treatment outcomes.

**Significance:**

Among pediatric patients with ALL/LL, significant disparities were found for specific comorbidities, particularly among females and younger patients who had higher rates of digestive tract diseases and infectious diseases. These findings are important as comorbidities can be considered in clinical decision-making in the management and treatment of these patients.

## Introduction

Acute lymphoblastic leukemia (ALL) is the most common pediatric malignancy (1, 2) and lymphoblastic lymphoma (LL) is the second most prevalent non–Hodgkin lymphoma in children and adolescents (3). ALL and LL are thought to exist on a spectrum of the same disease as they are biologically and clinically related malignancies that originate from immature lymphoid cells (4, 5). For example, patients with LL often exhibit low-level (5%–25%) bone marrow involvement, whereas those with ALL may present with lymphomatous disease (6, 7). Additionally, patients with LL can experience relapse with bone marrow involvement (>25% blasts) that meets the diagnostic criteria for ALL (7, 8). Recent studies have demonstrated improved outcomes for patients with LL treated with ALL-like regimens, leading to a shift toward ALL-based treatment approaches for LL (8–10).

Pediatric ALL and LL account for approximately 40% of childhood cancers in the United States and represent the second leading cause of cancer-related deaths in this age group (11). Although cure rates for childhood leukemia have dramatically improved over the past 40 years, these advances in treatment outcomes have not been equally realized across all sex, age, and racial/ethnic groups. Boys, patients diagnosed above 10 years of age, Hispanics, and non-Hispanic Blacks tend to experience poorer outcomes compared with females, those diagnosed at ≥10 years, and non-Hispanic Whites (NHW; refs. 12–17).

The disparity in outcomes may not be solely attributed to factors such as poor medication adherence or economic barriers (14, 18). For example, analyses restricted to patients with high medication compliance (≥90% adherence rate) and those controlling for known risk factors and economic variables still reveal that treatment outcomes for Hispanics and non-Hispanic Blacks are more than twice as poor as those for NHWs (19). These findings suggest that other factors may contribute to the racial and ethnic disparities in leukemia treatment outcomes.

Comorbidity, defined as the presence of one or more additional diseases alongside the primary disease, is known to affect treatment outcomes in adult cancers and other conditions (20–22). Thus, comorbidities could potentially be important in the sex, age, racial, and ethnic disparity in pediatric leukemia outcomes. However, the prevalence of major comorbidities among pediatric patients diagnosed with ALL/LL is not well characterized. It is imperative to expand current knowledge on the scope of comorbidities among pediatric patients with ALL/LL as an important first step to understanding the role of comorbidities in treatment outcomes and the development of targeted interventions to improve outcomes for these patients.

Given that ALL and LL share significant overlap in biological mechanisms, diagnostic criteria, therapeutic protocols, and treatment regimens, combining patients with these conditions in this study enables a more comprehensive understanding of comorbidities while ensuring clinical relevance. Accordingly, this study aims to determine the prevalence of comorbidities at diagnosis among pediatric patients with ALL/LL and examine if the prevalence varies by age, biological sex at birth, and race/ethnicity.

## Materials and Methods

Many studies of pediatric leukemia and lymphoma have been conducted using cooperative group cohorts or tumor registries (15, 23–25). Although these sources provide high accuracy in diagnostic classification, they reach only a limited and potentially biased group of patients as consortium data partially represent the total number of children with these conditions. Therefore, we used data from the TriNetX database, a federated health research network that provides access to cloud-based, Health Insurance Portability and Accountability Act–compliant, de-identified patient-level data from electronic health records (EHR). This platform represents broader populations and contains a wide range of clinical information. The data available in TriNetX include demographics, diagnoses, medications, laboratory tests, and procedures.

**Figure 1:**
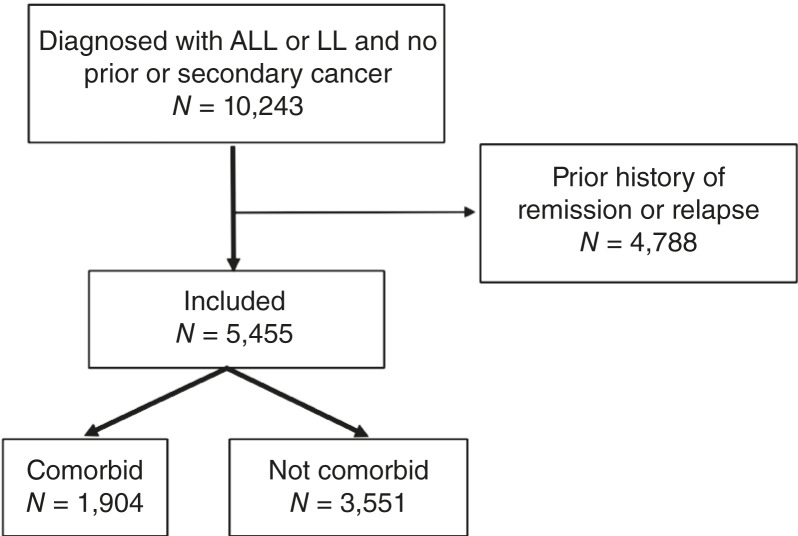
Flow diagram of cohort development. Pediatric patients with ALL/LL with no prior history of cancer, remission, or relapse before the index ALL/LL diagnosis were included.

We identified 10,243 pediatric patients from 81 healthcare organizations who were diagnosed with ALL/LL at ≤21 years of age between January 1, 2005, and June 30, 2020. Patients with a history of cancer or a secondary cancer diagnosis were excluded. After excluding 4,788 patients who had a diagnostic code for remission or relapse prior to their first diagnosis code for ALL/LL within the specified time frame (see Fig. 1), a total of 5,455 patients were included in the final study cohort. The identification of all diagnoses was based on phecodes, which are curated groupings of related billing codes from ICD-9-CM and ICD-10-CM. We categorized comorbidities of interest under the broad groups of pulmonary disease, cardiac disease, developmental disorders, vascular disease, cerebrovascular disease, immune disorders, metabolic disorders, infectious diseases, genitourinary disease, digestive tract disease, muscle and connective tissue disease, and nervous system disease (see Supplementary Table S1). Only comorbidities that were present (existing or newly occurred) within 3 months prior to the diagnosis of ALL/LL were included.

### Statistical analysis

Patient-level data were downloaded from TriNetX and analyzed using Python v3.10 and R 4.3.2. Baseline characteristics, including age at diagnosis, biological sex at birth, and race/ethnicity, of patients were summarized using count and percentages or median and IQR. The prevalence of comorbidities, with the corresponding 95% confidence intervals (CI), was calculated as the number of individuals with the comorbidity within a group (sex, age at diagnosis, and race/ethnicity) divided by the total number of patients within the group. Differences in prevalence between groups were compared using a two-proportion *z*-test.

### Data availability

The data analyzed in this study were retrieved on February 27, 2024, from the TriNetX Research Network, which provided access to electronic medical records from approximately 110 million patients from 95 healthcare organizations worldwide. For inquiries about the data, readers can contact TriNetX through the online form available at https://trinetx.com/. Further information is available from the corresponding author upon request.

## Results

**Figure 2:**
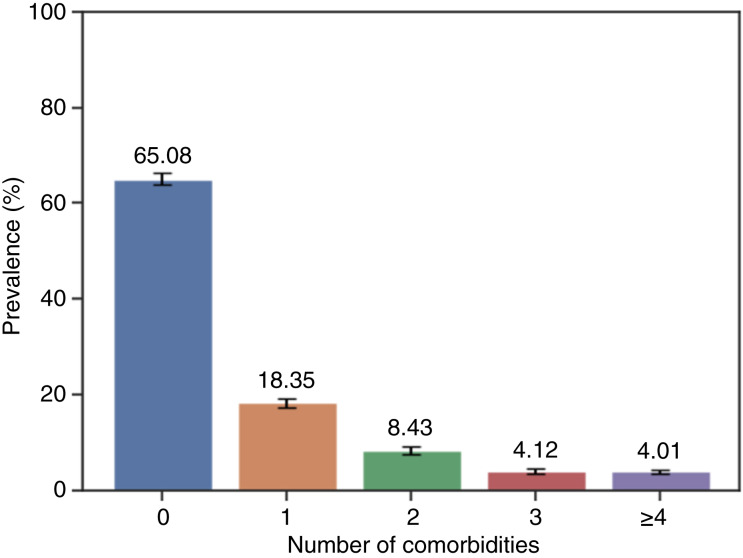
Prevalence of the number of comorbidities among pediatric patients with ALL/LL. Proportion of pediatric patients with ALL/LL with 0, 1, 2, 3, or ≥4 comorbidities present within 3 months before diagnosis. Comorbidities include pulmonary disease, cardiac disease, developmental disorders, vascular disease, cerebrovascular disease, immune disorders, metabolic disorders, infectious diseases, genitourinary disease, digestive tract disease, muscle and connective tissue disease, and nervous system disease. Error bars indicate 95% CI.

An overview of the demographic and clinical characteristics of the 5,455 unique participants is presented in Table 1. The majority of patients were male (*n* = 3,233, 59.3%) and White (3,233, 59.3%), with a median age at diagnosis of 9 years (range, IQR = 10). More than one third (*n* = 1,904, 34.1%) of patients were identified with at least one type of selected comorbidity within 3 months before their ALL/LL diagnosis (Fig. 2). Among these, 219 patients (4.01% of the entire cohort) had four or more comorbidities.

**Table 1:** Characteristics of the patient cohort

Characteristic	Overall (*N* = 5,455)
Median age at diagnosis (Q1, Q3)	9 (5, 15)
Biological sex at birth, *n* (%)	
Female	2,393 (43.9%)
Male	3,018 (55.3%)
Race, *n* (%)	
White	3,233 (59.3%)
Black or African American	393 (7.2%)
Other race	745 (13.7%)
Unknown	1,084 (19.9%)
Ethnicity, *n* (%)	
Hispanic or Latino	1,035 (19.0%)
Not Hispanic or Latino	2,971 (54.5%)
Unknown	1,449 (26.6%)
Initial diagnosis, *n* (%)	
ALL	4,863 (89.2%)
LL	208 (3.8%)
Lymphoid leukemia, unspecified	384 (7.0%)
Comorbidities	5,247 (96.2%)
Digestive	667 (12.2%)
Musculoskeletal	523 (9.6%)
Metabolic	513 (9.4%)
Neurologic	466 (8.5%)
Infectious	446 (8.2%)
Immune	334 (6.1%)
Genitourinary	280 (5.1%)
Cardiac	208 (3.8%)
Vascular	102 (1.9%)
Developmental	91 (1.7%)
Cerebrovascular	34 (0.6%)
Pulmonary	18 (0.3%)

### Prevalence of comorbidities by type

**Figure 3:**
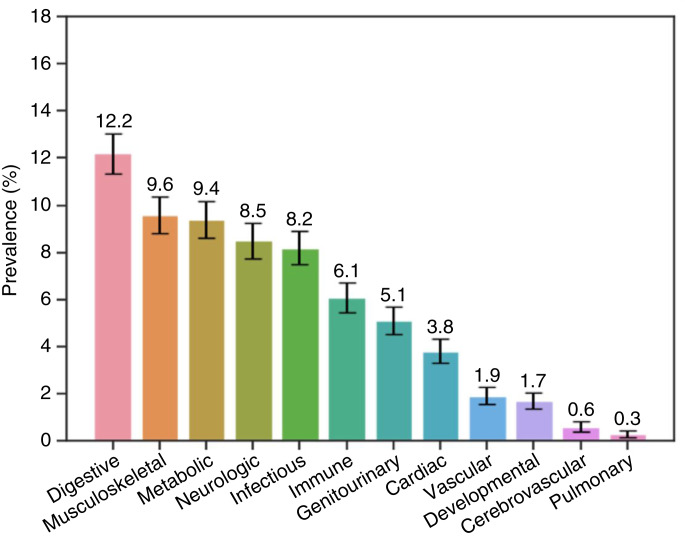
Prevalence of comorbidity groups among pediatric patients with ALL/LL. Proportion of pediatric patients with ALL/LL with different groups of comorbidities. Error bars indicate 95% CI.

The prevalence of each category of selected comorbidities identified within 3 months prior to their ALL/LL diagnosis among pediatric patients is shown in Fig. 3. Digestive tract disorders were the most common comorbidity identified, with 667 patients identified within 3 months prior to their diagnosis of ALL/LL(12.2%; 95% CI, 11.4%–13.1%), followed by muscle and connective tissue disease (*n* = 523, 9.6%; 95% CI, 8.8%–10.4%), metabolic disorders (*n* = 513, 9.4%; 95% CI, 8.6%–10.2%), nervous system disease (*n* = 466, 8.5%; 95% CI, 7.8%–9.3%), infectious disease (*n* = 446, 8.2%; 95% CI, 7.4%–8.9%), and immune disorders (*n* = 334, 6.1%; 95% CI, 5.5%–6.8%). Approximately 5% of participants (*n* = 280, 5.1%; 95% CI, 4.6%–5.7%) had a documented genitourinary disease. Cardiac, vascular, cerebrovascular, and pulmonary disease and developmental disorders each had a prevalence ≤5%.

### Prevalence of comorbidities by sex

**Figure 4:**
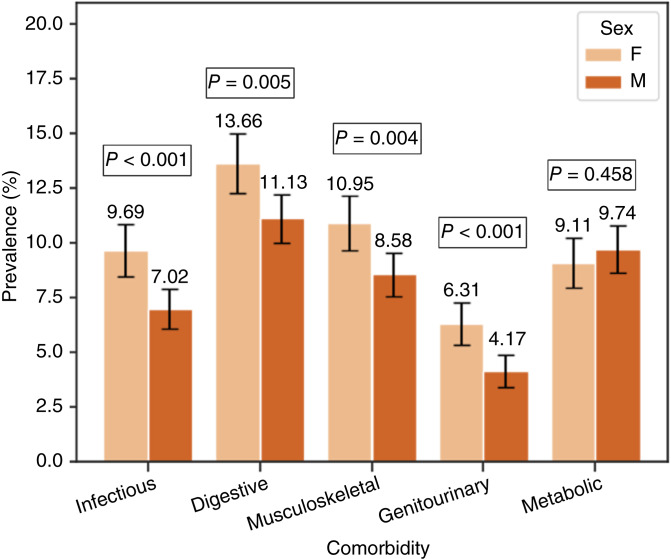
Prevalence of comorbidity by biological sex at birth among pediatric patients with ALL/LL. Proportion of female and male pediatric patients with ALL/LL with comorbidities. Error bars indicate 95% CI. F, female; M, male.

The overall comorbidity prevalence for female patients (36.6%) was slightly higher than male patients (*n* = 33.6%), and the difference was statistically significant (3.00%; 95% CI, 0.38%–5.6%; *P* = 0.024; Table 2). To further elucidate this sex-based difference, the prevalence of specific comorbidities was analyzed (Fig. 4). The greatest sex disparity was observed for infectious diseases, with females exhibiting a higher prevalence of 9.4% compared with 6.8% in males (*P* < 0.001). This was followed by digestive tract disease, in which the prevalence was 13.7% in females versus 11.1% in males (*P* = 0.005), musculoskeletal diseases (11.0% in female, 8.6% in male; *P* = 0.004), and genitourinary conditions, with 6.3% in females compared with 4.2% in males (*P* < 0.001). Conversely, males exhibited a slightly higher prevalence of metabolic disorders than females (9.5% vs. 8.8%), but this difference was not statistically significant (*P* = 0.46). Comparison of the other comorbidities did not reveal statistically significant differences between the groups (all *P* > 0.05).

**Table 2:** Prevalence of overall comorbidity by sex, age at diagnosis, and race/ethnicity

Overall comorbidity	Groups	*N*	Comorbidity (*n*, %)	Difference	95% CI	*P* value^a^
Biological sex at birth	Female	2,393	876 (36.6%)	3.00%	0.38%, 5.6%	0.024
	Male	3,018	1,015 (33.6%)			
Age at diagnosis	>10	2,473	865 (35.0%)	0.14%	−2.4%, 2.7%	0.940
	≤10	2,982	1,039 (34.8%)			
Race/ethnicity	NHW	2,193	823 (37.5%)	4.40%	1.8%, 7.0%	<0.001
	Others	3,262	1,081 (33.1%)			

a Two-sample *Z*-test for proportion.

### Prevalence of comorbidities by age at diagnosis

**Figure 5:**
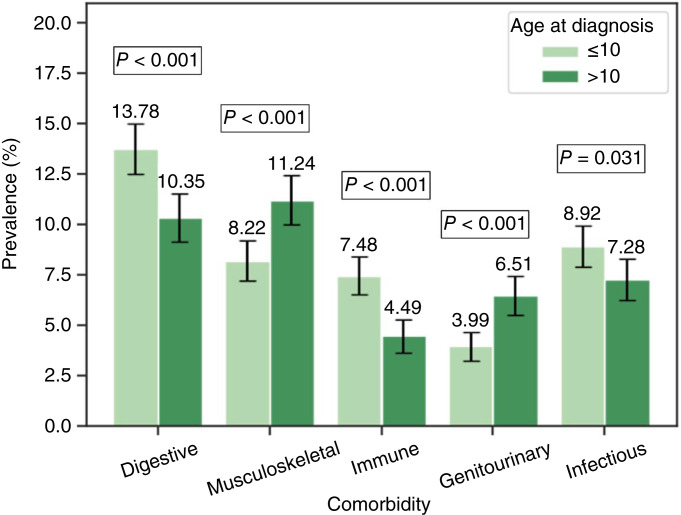
Prevalence of comorbidity by age at diagnosis among pediatric patients with ALL/LL. Proportion of pediatric patients with ALL/LL with comorbidities diagnosed at a younger (≤10 years) or older age (>10 years). Error bars indicate 95% CI.

In this entire cohort, more than half (*n* = 2,982, 54.7%) of the patients were diagnosed with ALL/LL at ≤10 years of age. The prevalence of overall comorbidity for those ≤10 years (34.8%) was comparable with those >10 years (35.0%), *P* = 0.94 (Table 2). However, statistically significant differences were observed for specific comorbidities (Fig. 5), with younger patients (≤10 years) having a higher prevalence of digestive tract diseases (13.8% vs. 10.4%; *P* < 0.001), immune disorders (7.5% vs. 4.5%; *P* < 0.001), and infectious diseases (8.9% vs. 7.3%; *P* = 0.031). Conversely, older patients (>10 years) exhibited a higher prevalence of musculoskeletal disease (11.2% vs. 8.2%; *P* < 0.001) and genitourinary disease (6.5% vs. 4.0%; *P* < 0.001) compared with their younger counterparts. Comparison of the other comorbidities did not reveal statistically significant differences between the groups (all *P* > 0.05).

### Prevalence of comorbidities by race and ethnicity

**Figure 6:**
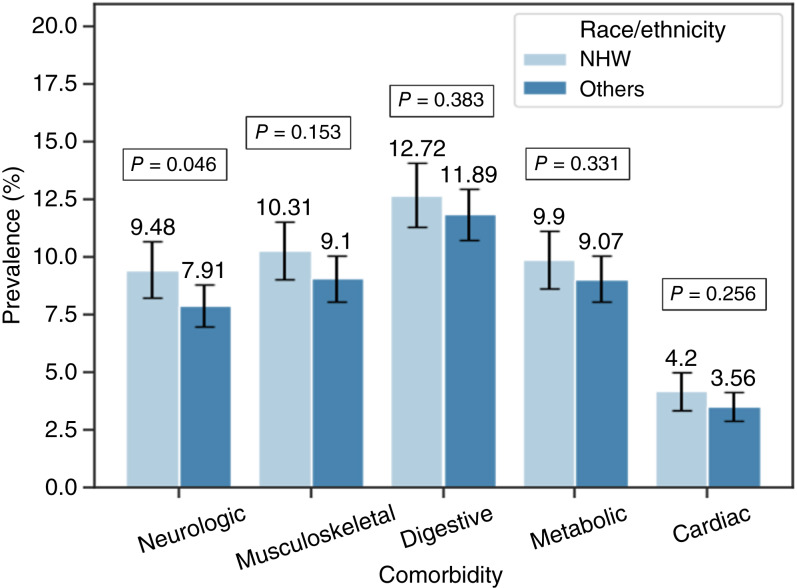
Prevalence of comorbidity by race/ethnicity among pediatric patients with ALL/LL. “Others” includes mostly African Americans, Hispanics, and Asians. Error bars indicate 95% CI.

Due to the limited number of patients in other races and ethnicity, the analysis of comorbidity by race/ethnicity was performed between NHW patients and the rest of the cohort, labeled as “others,” revealing a difference (37.5% vs. 33.1%; difference 4.40%; 95% CI, 1.8%–7.0%; *P* < 0.001) in overall comorbidity prevalence between the two groups (Table 2). For each specific comorbidity, NHWs consistently exhibited higher prevalence than the other group, but the differences were not statistically significant (Fig. 6). The most substantial disparity was observed for nervous system disease, with a prevalence of 9.5% among NHW patients compared with 7.9% among others (*P* = 0.046). Comparison of the other comorbidities did not reveal statistically significant differences between the groups (all *P* > 0.05).

## Discussion

Previous studies have broadly examined the impact of comorbidities on cancer outcomes and treatment, including quality of life in survivors of ALL (21, 26). However, to the best of our knowledge, this is the first study to compare the prevalence of specific comorbidities across biological sexes at birth, age groups, or race and ethnicity groups in pediatric patients diagnosed with ALL/LL. Our analysis including 5,455 unique pediatric patients with ALL/LL in the TriNetX Research Network revealed that about one third of patients with ALL/LL had at least one comorbidity with digestive, musculoskeletal, and metabolic diseases as the most predominant comorbidities. Age and sex disparities in prevalence were also observed. Younger patients had a higher prevalence of digestive tract, infectious, and immune diseases compared with older patients who had a higher prevalence of musculoskeletal disease and genitourinary disease. The analysis also revealed that the prevalence of overall comorbidity, digestive tract, musculoskeletal, genitourinary, and infectious disease were higher in females than males.

The observed overall prevalence of comorbidities in the pediatric patients with ALL/LL in our study is lower than that (∼75%) reported in adult patients with cancer (27). This difference is expected as adults tend to have more comorbidities because of aging, shared risk factors for other diseases, and underlying biological mechanisms (28).

One of the most unevenly distributed comorbidities was digestive tract disease, with a 2.5% higher prevalence in females compared with males and 3.4% higher in younger (≤10 years) than older patients. A closer look at digestive tract disorders included in our analysis revealed constipation as the most common diagnosis (Supplementary Fig. S1). A recent study identified younger age (<6 years) and female sex as significant predictors of constipation in pediatric patients with ALL during induction therapy (29). Multivariate logistic regression in that study revealed that children below 6 years of age were more likely to experience constipation than those ≥12 years of age [OR = 1.32 (95% CI, 1.13–1.55); *P* < 0.001], with female sex also associated with slightly increased odds [OR = 1.16 (95% CI, 1.02–1.31); *P* = 0.024]. Similarly, a higher prevalence of constipation has been observed in pediatric females in noncancer populations (30). Although the underlying mechanisms remain unclear, further research is needed to investigate the relationship among constipation, ALL/LL treatment, and outcomes in children.

The observed age and sex disparity was also observed for genitourinary diseases (2.1% higher in females and 2.5% higher in older patients). Renal failure was the most common genitourinary condition with urinary tract infections among the top 10 diagnoses (Supplementary Fig. S2). Although acute renal failure has been a known complication after chemotherapy initiation in ALL, it rarely presents as a primary manifestation of the disease. Case reports have documented nephromegaly and hypertension at ALL diagnosis (31, 32). An update of the European Association of Urology guidelines demonstrated a higher incidence of urinary tract infections in females over 6 months, largely due to anatomic differences (33, 34). Furthermore, a recent study suggests that the higher prevalence of both digestive and genitourinary conditions in females may be due to shared developmental origins, anatomical proximity, and neural cross-talk between pelvic organs (35). It is, however, not clear why musculoskeletal and infectious diseases are more prevalent in younger patients compared with older patients or why infectious and immune diseases are more prevalent in younger than older patients.

This study has several limitations. Comorbidities were obtained within 3 months before diagnosis, and these comorbidities could have potentially arisen because of the cancer. Nevertheless, the findings are still important as these comorbidities will be considered in treatment decision-making, despite the cause or timing of diagnosis. Additionally, less severe comorbidities may be underreported. Potential inaccuracies in diagnosis dates due to vague coding and limited auxiliary information in the EHR should also be acknowledged. Moreover, comorbidities were identified based on a single record in the EHR, which could lead to misclassification. Future research should employ more stringent criteria for diagnosing comorbidities and incorporate procedure and laboratory data to further explore contributing factors. Although our study showed a higher prevalence of overall comorbidity and specific comorbidities for NHWs compared with all other races/ethnicities combined, a larger study separating the different race/ethnicity groups is warranted to provide further insights into the variation of comorbidities by race/ethnicity among pediatric patients with ALL/LL. In adults, the prevalence of comorbidities is higher among Native Americans and African Americans than other race/ethnicity groups (36). However, a study of adolescents showed that depending on the comorbid condition, the prevalence may be higher or lower in one racial/ethnic group compared with others (37). Lastly, we did not separate congenital immune disorders (also called inborn errors of metabolism) from autoimmune disorders. Congenital disorders are more common in males and present at a younger age. Autoimmune disorders are more common in older female children (especially preteens and teenagers). In addition, congenital immune disorders are thought to predispose children to cancer (so they might not necessarily be a distinct comorbidity but more of a continuum in diagnosis with known complication), whereas autoimmune disorders are distinct entities and can be considered true unrelated comorbidity. Nevertheless, their presence at diagnosis will still be important in making treatment decisions and potentially affect survival/outcome.

In conclusion, in this study, we examined the prevalence of comorbidities among pediatric patients with ALL/LL, focusing on variations by biological sex at birth, age at diagnosis, and race/ethnicity. Although overall comorbidity prevalence varied minimally across these groups, significant disparities were found for specific comorbidities, particularly among females and younger patients who had higher rates of digestive tract diseases and infectious diseases. These findings are important as comorbidities can influence cancer management and treatment options. Our study provides information about the prevalence of specific comorbidities in different groups of pediatric patients with ALL/LL that can be used for clinical decision-making. Further studies are necessary to determine if these disparities contribute to differences in treatment outcomes.

## Supplementary Material

Supplementary Table S1 Supplementary Table S1 shows the ICD-9/10-CM codes for used for identifying patients with ALL/LL and comorbidities

Supplementary Figure S2 Top 10 common diagnosis in genitourinary diseases (with each diagnosis code counted once per patient within the 3 months prior to their ALL/LL diagnosis, regardless of multiple occurrences)

Supplementary Figure S1 Top 10 common diagnosis in digestive tract disorders (with each diagnosis code counted once per patient within the 3 months prior to their ALL/LL diagnosis, regardless of multiple occurrences)
