# Supplementary material for: Age and Sex Differences in the Prevalence of Specific Comorbidities among Patients with Pediatric Acute Lymphoblastic Leukemia and Lymphoblastic Lymphoma at Diagnosis
Source: Cancer Res Commun. 2025 Apr 1;5(4):549–55. doi: 10.1158/2767-9764.CRC-24-0517 (PMC11961403; doi:10.1158/2767-9764.CRC-24-0517)
Supplement: Supplementary Table S1 — shows the ICD-9/10-CM codes for used for identifying patients with ALL/LL and comorbidities [file crc-24-0517_supplementary_table_s1_suppst1.docx]

Supplementary Table S1. ICD-9/10-CM codes used for developing the cohort

| Condition | ICD-10-CM | ICD-9-CM |
| --- | --- | --- |
| ALL or LL Diagnosis | C91.00, C91.90, C91.Z0, C83.5X | 204.00, 204.90, 204.20, 204.80, 200.1x |
| ALL or LL remission | C91.01, C91.91, C91.Z1 | 204.01, 204.91, 204.21, 204.81 |
| ALL or LL relapse | C91.02, C91.92, C91.Z2 | 204.02, 204.92, 204.22, 204.82 |
| Pulmonary | I26-I28 | 415-417 |
| Cardiac | I30-I52 | 420-429  420 Acute pericarditis  421 Acute and subacute endocarditis  422 Acute myocarditis  423 Other diseases of pericardium  424 Other diseases of endocardium  425 Cardiomyopathy  426 Conduction disorders  427Cardiac dysrhythmias  428 Heart failure  429 Ill-defined descriptions and complications of heart disease |
| Cerebrovascular | I60-I69 | 430-438 |
| Vascular | I70-I89  Diseases of arteries, arterioles and capillaries, Diseases of veins, lymphatic vessels and lymph nodes, not elsewhere classified | 440-459  diseases of arteries, arterioles, and capillaries (440-449), diseases of veins and lymphatics, and other diseases of circulatory system (451-459) |
| Developmental Disorders | F80-89 | 315 Specific delays in development |
| Immune Disorders | D80-89 | 279 Disorders involving the immune mechanism |
| Metabolic Disorders | E70-88 | 270-277 |
| Infectious | A00-99, B00-99 | 001-139  Infectious and parasitic diseases |
| Genitourinary | N00-99 | 580-629  Diseases of the genitourinary system |
| Digestive | K00-99 | 520-579  Diseases of the digestive system (520-579) |
| Musculoskeletal | M00-99 | 710-739  Diseases of the musculoskeletal system and connective tissue (710-739) |
| Neurological | G00-99 | 320-389  Diseases of the nervous system and sense organs |
