# Supplementary material for: Age and Sex Differences in the Prevalence of Specific Comorbidities among Patients with Pediatric Acute Lymphoblastic Leukemia and Lymphoblastic Lymphoma at Diagnosis
Source: Cancer Res Commun. 2025 Apr 1;5(4):549–55. doi: 10.1158/2767-9764.CRC-24-0517 (PMC11961403; doi:10.1158/2767-9764.CRC-24-0517)
Supplement: Supplementary Figure S2 — Top 10 common diagnosis in genitourinary diseases (with each diagnosis code counted once per patient within the 3 months prior to their ALL/LL diagnosis, regardless of multiple occurrences) [file crc-24-0517_supplementary_figure_s2_suppsf2.pptx]

## Slide 1
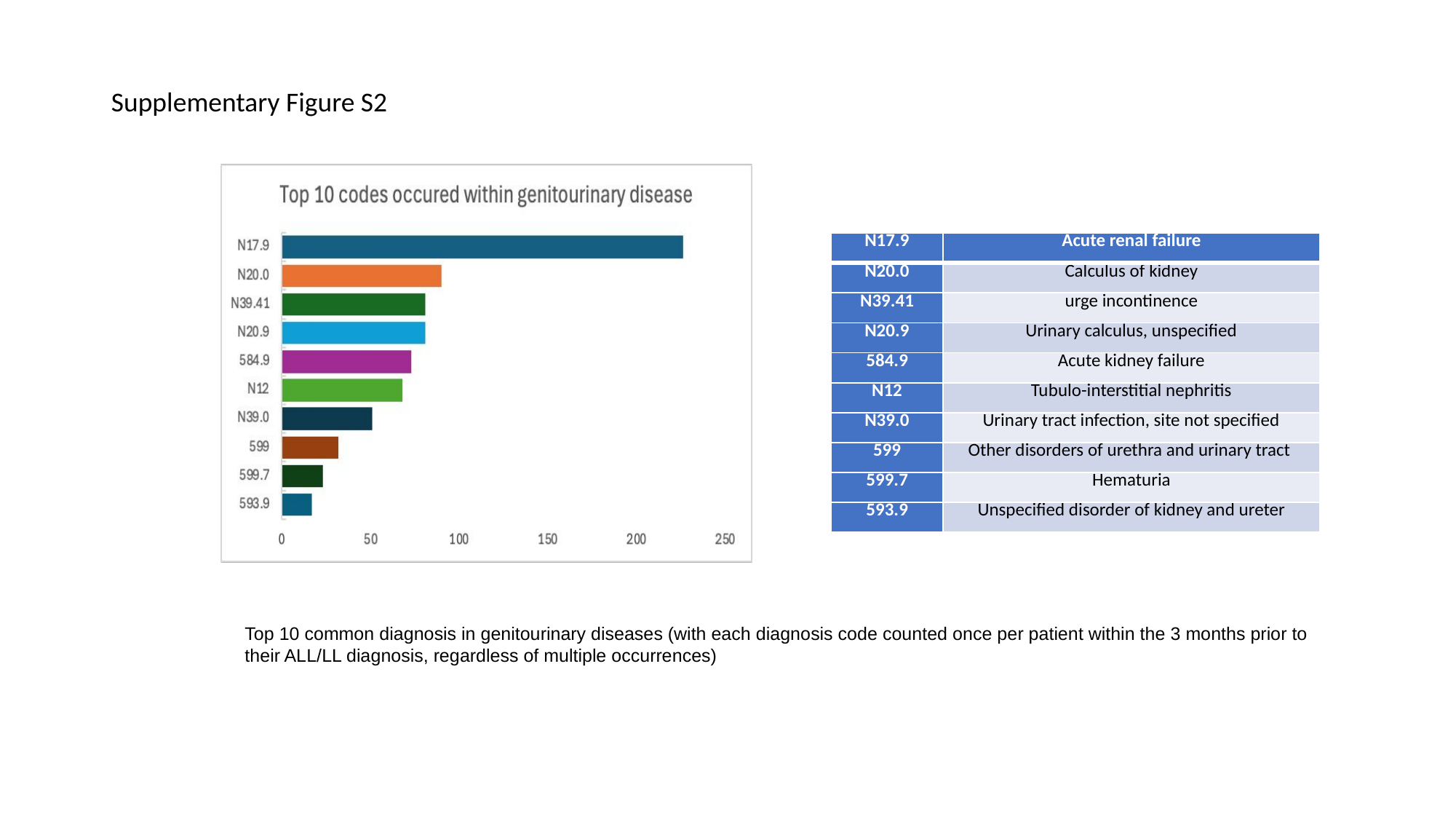

Supplementary Figure S2
| N17.9 | Acute renal failure |
| --- | --- |
| N20.0 | Calculus of kidney |
| N39.41 | urge incontinence |
| N20.9 | Urinary calculus, unspecified |
| 584.9 | Acute kidney failure |
| N12 | Tubulo-interstitial nephritis |
| N39.0 | Urinary tract infection, site not specified |
| 599 | Other disorders of urethra and urinary tract |
| 599.7 | Hematuria |
| 593.9 | Unspecified disorder of kidney and ureter |
Top 10 common diagnosis in genitourinary diseases (with each diagnosis code counted once per patient within the 3 months prior to their ALL/LL diagnosis, regardless of multiple occurrences)
