# Supplementary material for: Age and Sex Differences in the Prevalence of Specific Comorbidities among Patients with Pediatric Acute Lymphoblastic Leukemia and Lymphoblastic Lymphoma at Diagnosis
Source: Cancer Res Commun. 2025 Apr 1;5(4):549–55. doi: 10.1158/2767-9764.CRC-24-0517 (PMC11961403; doi:10.1158/2767-9764.CRC-24-0517)
Supplement: Supplementary Figure S1 — Top 10 common diagnosis in digestive tract disorders (with each diagnosis code counted once per patient within the 3 months prior to their ALL/LL diagnosis, regardless of multiple occurrences) [file crc-24-0517_supplementary_figure_s1_suppsf1.pptx]

## Slide 1
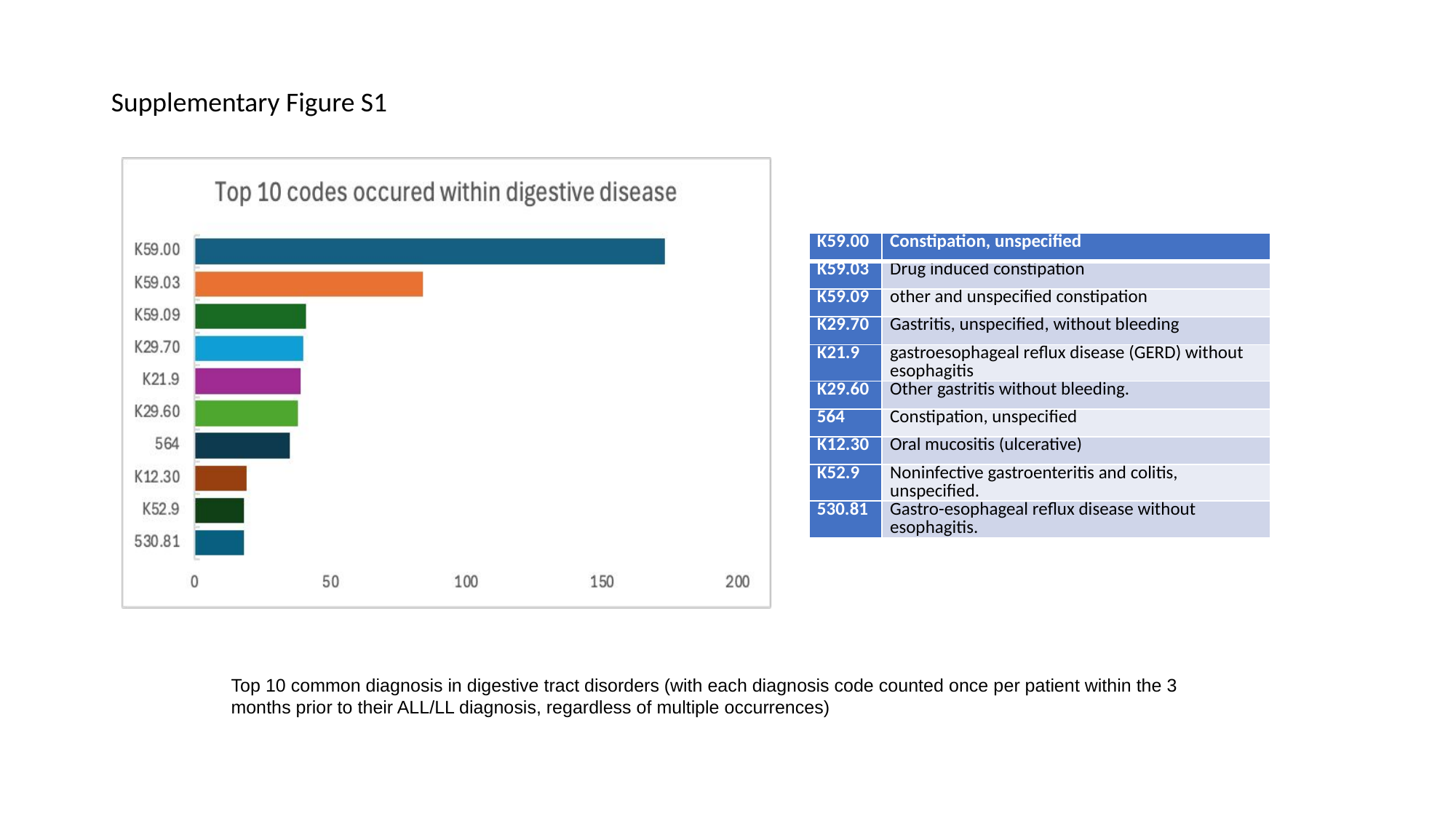

Supplementary Figure S1
| K59.00 | Constipation, unspecified |
| --- | --- |
| K59.03 | Drug induced constipation |
| K59.09 | other and unspecified constipation |
| K29.70 | Gastritis, unspecified, without bleeding |
| K21.9 | gastroesophageal reflux disease (GERD) without esophagitis |
| K29.60 | Other gastritis without bleeding. |
| 564 | Constipation, unspecified |
| K12.30 | Oral mucositis (ulcerative) |
| K52.9 | Noninfective gastroenteritis and colitis, unspecified. |
| 530.81 | Gastro-esophageal reflux disease without esophagitis. |
Top 10 common diagnosis in digestive tract disorders (with each diagnosis code counted once per patient within the 3 months prior to their ALL/LL diagnosis, regardless of multiple occurrences)
